# The Continued Threat of Tuberculosis

**DOI:** 10.3201/eid0811.020468

**Published:** 2002-11

**Authors:** Tom Navin, Scott McNabb, Jack T. Crawford

**Affiliations:** * Centers for Disease Control and Prevention, Atlanta, Georgia, USA

Why would a journal that tracks and analyzes emerging infectious disease trends devote an entire issue to tuberculosis, a disease that emerged some 15,000 to 35,000 years ago (1,2)? The disturbing answer is that tuberculosis is reappearing in many countries as a public health crisis. Thus, if not an emerging disease, it is an important reemerging disease, and though ancient, it is not a disease of the past. A staggering 1.9 million around the globe die of tuberculosis each year—another 1.9 billion are infected with *M. tuberculosis* and are at risk for active disease (3).

In the 20th century, the United States made impressive strides in tuberculosis control. From the early 1900s, when some areas began systematic reporting of death rates, tuberculosis rates steadily declined from approximately 200 deaths per 100,000 per year to less than 1 death per 100,000 in 1985. In 1953, a national surveillance system was established for reporting new cases of tuberculosis disease; that year, reported annual incidence was 53 cases per 100,000 population (4). From 1953 to 1984, tuberculosis disease incidence dropped steadily at an average rate of 5.8% per year to 9.4 cases per 100,000.

In 1985, however, the United States saw a reversal in this long-standing downward trend, and tuberculosis reemerged as a public health threat. From 1985 to 1992, not only did the number of cases increase from 22,201 to 26,673, but also large outbreaks were reported. Many of these, especially in hospitals and other health-care settings in large cities (5), were caused by multidrug-resistant *M. tuberculosis*. Several factors contributed to this increase, including the emergence of the HIV epidemic and large influxes of immigrants from countries in which tuberculosis was common. Perhaps the major reason for the reemergence, however, was the end in 1972 of categorical federal funding for control activities and the subsequent deterioration of public health infrastructure for tuberculosis.

In response to the crisis of reemerging tuberculosis, categorical grants were restored and federal funding was increased. The funding, modest at first, rose sharply in 1992 and again in 1993 and 1994. The Centers for Disease Control and Prevention (CDC) transfers most of its appropriated funds to tuberculosis control programs in states and large cities through cooperative agreements. These funds support clinics and laboratories, administer directly observed therapy, intensify investigation of latent infection in persons at high risk for active disease, sponsor clinical and epidemiologic research, and expand surveillance to monitor the impact of these efforts. Renewed investments paid off, and after a peak in 1992, tuberculosis incidence in the United States has declined each year. From 1992 to 2001, the annual decline averaged 7.3%, even greater than before 1985. But future success is not guaranteed. The National Academy of Sciences Institute of Medicine, in its 2000 report on tuberculosis control efforts in the United States, warned against the “complacency and neglect” that come with declining numbers of cases and reaffirmed the goal of TB elimination (annual incidence of >1 case per 1,000,000 population) in the United States (6).

In 2001, the 15,989 tuberculosis cases reported to CDC represented only a 2% decline from 2000, the smallest decline in 9 years. Although data from a single year do not constitute a trend, these numbers may be the first sign of stagnation in our control efforts. The proportion of cases in persons born outside the United States is growing; in 2001, that figure reached 50%. Efforts to reduce tuberculosis transmission in the United States have little effect on reducing risk for those infected elsewhere. The proportion of cases in persons born in other countries will probably continue to rise, unless domestic programs providing tuberculosis services for immigrants are strengthened and international programs are expanded. Another risk, in the current climate of bioterrorism, is the possible intentional spread of multidrug-resistant *M. tuberculosis.* This risk requires new tools for detection and rapid and effective response. Currently strengthened surveillance systems closely monitor changes in disease epidemiology. If tuberculosis elimination progress in the United States slows, we are prepared to respond quickly.
